# COVID-19 and Seasonal Influenza: No Room for Two

**DOI:** 10.7759/cureus.18007

**Published:** 2021-09-15

**Authors:** Nikolaos Spantideas, Anastasia M Bougea, Eirini G Drosou, Neha Khanderia, Summina Rai

**Affiliations:** 1 ENT, Northwick Park Hospital, London, GBR; 2 Neurology, Aeginition Hospital, National and Kapodistrian University of Athens, Athens, GRC; 3 Speech Therapy, Athens Speech Language and Swallowing Institute, Athens, GRC

**Keywords:** twindemic, seasonal influenza, covid-19, sars-cov-2, pandemic, epidemiology, flunet, cdc, fluview, ecdc

## Abstract

Objective

With the outbreak of COVID-19 in December 2019 fears were expressed for a possible twindemic in the coming flu seasons. Fortunately, this was not the case for the 2019-2020 and 2020-2021 flu seasons as flu showed very low historical rates during these periods. The objective of our study was to look at the existing flu data for the 2019-2021 period and analyze possible reasons for the near absence of seasonal flu.

Methods

We performed retrospective surveillance regarding seasonal influenza rates for the years 2019-2021, the years that the COVID-19 was present. Epidemiological data concerning seasonal influenza for the years 2019-2021 were collected and analyzed

Results

Extremely low numbers of flu cases were reported in FluNet, FluView, and TESSy influenza surveillance systems during the years 2019, 2020, and 2021 compared to previous years prior to COVID-19.

Conclusions

A twindemic outbreak during the 2019-2021 flu seasons did not occur despite expressed concerns. The worldwide implementation of mitigation measures for individuals and communities to control severe acute respiratory syndrome coronavirus 2 (SARS-CoV-2) transmission, the increased flu vaccination rate, the virus interference and the lower rate of testing for flu are the main reasons for the marked decrease in reported flu cases during 2019-2021 flu seasons.

## Introduction

Seasonal influenza is a worldwide contagious upper and lower respiratory tract illness caused predominantly by type A and B influenza viruses. Influenza epidemics have a seasonality generally occurring in regular annual epidemics in autumn and winter. In the northern hemisphere, it occurs between November and April (with a peak in December-February) and in the southern hemisphere between May and September (peak July-August) [1]. Influenza epidemics usually occur annually and influenza pandemics every 10-15 years.

Type A viruses are responsible for the highest burden of disease during seasonal epidemics and type B infections are less common and usually milder than influenza A. The World Health Organization (WHO) estimates that globally seasonal influenza epidemics cause an annual three to five million severe cases and 290,000 to 650,000 deaths. Pregnant women, young children, the elderly, and people with chronic illnesses are at high risk for severe illness and death associated with influenza virus infection [2]. The economic and health care burden of influenza epidemics is substantial despite the short duration of the illness [3].

In December 2019, in Wuhan, a cluster of pneumonia of unknown cause was reported. In early January 2020, Chinese scientists identified a new type of coronavirus and shared its genetic sequence. On March 11, 2020, the rapid increase in the number of cases outside China led the WHO to characterize this outbreak as the coronavirus disease 2019 (COVID-19) pandemic [4]. The virus that causes COVID-19 was named severe acute respiratory syndrome coronavirus 2 (SARS‑CoV‑2).

During winter and spring 2019-2020 in the northern hemisphere, the influenza season overlapped with the first wave of COVID-19. In autumn and winter of 2020-2021, seasonal influenza was expected to overlap with the second and third waves of the COVID-19 pandemic. Experts feared that an average flu season compounded by the spread of SARS‑CoV‑2 could lead to a dual outbreak, overwhelming the health care system and limiting the ability of hospitals to treat patients. Health care systems had to be prepared to face the same numbers of hospitalizations and deaths from seasonal flu this year as in previous years.

Fortunately, according to current data, this does not appear to have transpired as the number of flu cases seems to be at a historically low level for the 2020-2021 autumn-winter period. It is, however, not the only influenza that disappeared over the last year. There were also substantial drops in other respiratory illnesses, including the respiratory syncytial virus which is the most common cause of pneumonia in infants. Due to the similarities in transmission methods between influenza and SARS-CoV-2, epidemiologists believe that the public health measures taken to keep the coronavirus from spreading also stopped the spread of flu.

Although the WHO and other health agencies warn that the data on seasonal influenza could be less reliable than in past years as we focused on the COVID-19 pandemic, several metrics provide an additional indication that the existing seasonal influenza burden is actually low.

Several reasons have been proposed to explain the very low incidence of flu during the COVID-19 pandemic. All factors that could contribute to the disappearance of flu are under investigation and the answers to these critical questions will help us to better organize our strategy not only for the COVID-19 pandemic but also for future pandemics. The scope of this study was to analyze existing world data for the seasonal flu and look for possible reasons for the extremely low seasonal flu cases during the COVID-19 pandemic.

## Materials and methods

We conducted a retrospective open access database analysis of laboratory-confirmed influenza for the years 2019, 2020, and 2021 (the years following the start of the COVID-19 pandemic).

The influenza data for the southern hemisphere were derived from the World Health Organization FluNet (WHO Influenza Surveillance Report - known as FluNet) [5,6]. The data were submitted by National Influenza Centers (NICs) of the Global Influenza Surveillance and Response System (GISRS) and other national influenza reference laboratories collaborating actively with GISRS. The southern hemisphere influenza data reported to the WHO’s FluNet platform are usually coming from three southern hemisphere countries - Oceania (Australia), South America (Chile), and southern Africa (South Africa) - that serve as robust sentinel sites for influenza.

The USA influenza data were downloaded from the Center for Disease Control and Prevention FluView (CDC Weekly US Influenza Surveillance Report - known as FluView) [7,8]. FluView data come from both US WHO collaborating laboratories and the National Respiratory and Enteric Virus Surveillance System (NREVSS). The CDC collects data from approximately 300 US clinical laboratories located throughout all 50 states and uses a mathematical model to estimate influenza morbidity and mortality (numbers of influenza illnesses, medical visits, hospitalizations, and deaths in the United States). The impact of influenza vaccination on these numbers is included in the final estimation.

The European influenza data were downloaded from the Center for Disease Prevention and Control (The European Surveillance System - known as TESSy) [9,10]. The European Centre for Disease Prevention and Control (ECDC) and the WHO Regional Office for Europe jointly report on the influenza situation in the wider European region which covers 53 countries of the WHO European Region (including the European Union/European Economic Area {EU/EEA} Member States). The weekly influenza-positive results from the southern hemisphere, Europe, and the USA were summarized and analyzed.

## Results

Globally, for the period between May 2019 to April 2020 and May 2020 to April 2021, there was a significant reduction in the positivity rate according to the WHO influenza update reports, No. 342-391 (Table 1) [5].

**Table 1:** Total tests, positive tests, and positivity rate of influenza for the period from May 2019 to March 2021

Period (month/year)	Total tests	Positive tests	Positivity rate
May 2019 to March 2020	2.458.076	408.613	16,6%
May 2020 to March 2021	4.829.789	5.467	0,11%

Northern hemisphere

In Europe, across the WHO European Region, there was a significant reduction in reports of detected influenza cases during the influenza seasonal period for 2021 compared to similar periods between 2020 and 2019 (Table 2) [9-11].

**Table 2:** Influenza cases reported to the European Surveillance System for the years 2019-2021

Year (April reports)	Flu detected cases
2019	203.585
2020	162.345
2021	758

According to the CDC Weekly US Influenza Surveillance Report, clinical laboratories in the United States reported 1766 positive results out of 931,726 specimens tested (just 0.2%) from the start of the current flu season in September 2020 up until week 20 2021 [7]. In contrast, the CDC reported about 250,000 positive specimens out of 1.5 million tested in the 2019-2020 flu season [8]. Public health laboratories reported 243 positive specimens out of 438,098 tested. 

The hospitalization rate for the 2020-2021 flu season in the United States was just 0.7 per 100,000 people, the lowest since the agency started tracking flu data in 2005. In fact, over the course of the last flu season, there was just one pediatric flu death compared to 196 in the 2019-2020 flu season.

Southern hemisphere

According to the WHO Weekly Influenza Update for July 2020, seasonal influenza activity in the southern hemisphere remained extremely low compared to what would be expected based on historical trends. In fact, no region is reporting more than 10% test positivity for seasonal influenza and several regions, including South and Southeast Asia and parts of South America and Africa, reported no influenza cases. For comparison, most regions were reporting more than 10% test positivity at the same time in 2019, including several that reported test positivity greater than 30%. The southern hemisphere influenza season typically peaks around this time of year, but the WHO stated that “the influenza season has not commenced” (Table 3) [6].

**Table 3:** Southern hemisphere documented flu cases, April through mid-August 2020

Country	2018	2019	2020
Australia	925	9933	33
South Africa	711	1094	6
Argentina	1517	4623	53
Chile	2439	5007	12

## Discussion

For the last 30 years, the numbers of flu infections have been relatively stable, but during 2020 and 2021, seasonal flu practically disappeared. The global decline in influenza virus circulation during 2020-2021 coincided with two major events, the outbreak of the COVID-19 pandemic and the use of individual and community mitigation measures for COVID-19 pandemic control. The recommended mitigation measures for the COVID-19 pandemic are likely to have been effective in reducing the incidence and impact of influenza, and some of these mitigation measures could have a role in preventing influenza in future seasons. It is difficult to separate the effect that individual and community mitigation measures might have had on influenza transmission and we do not yet know which public health measures were most effective in eradicating the flu during 2020-2021 flu seasons. The real role and contribution of each measure for flu prevention must be studied in order to understand its significance and impact on influenza control. Data from the current pandemic might help to answer critical questions, not only regarding the effect of community mitigation measures on the transmission of influenza during the 2020 and 2021 flu seasons but also for other pandemics that might arise in the future.

Among the reasons that have been proposed to explain the very low incidence of flu cases during the COVID-19 pandemic are (1) the worldwide implementation of individual and community mitigation measures for SARS-CoV-2 transmission control. Influenza and SARS-CoV-2 are both primarily transmitted in much the same way via respiratory droplets. So, it is reasonable to assume that the non-pharmaceutical interventions (NPIs) that are effective against COVID-19 would also be effective against the seasonal flu and even more so given the lower R-value due to pre-existing population immunity. The lower transmissibility of seasonal influenza virus (R0 = 1.5) compared with that of SARS-CoV-2 (R0 = 2.5) likely contributed to a more substantial interruption in influenza transmission [12]. Alongside wearing face masks and maintaining social distancing, travel restrictions, the collapse of traveling restricted transmission from country to country. With children acting as the main reservoir of these viruses, closing schools also seems to contribute immensely to reduced spread across the community [13]. (2) An increased number of people were vaccinated this year under the threat of co-infection by two different viruses - the flu virus and the SARS-CoV-2 virus. Seasonal influenza vaccination is considered the most effective way to prevent influenza virus infection and its associated complications. In the USA alone there were about 20 million more vaccinations than in past years. In New York, there was a 37% increase in the number of adults aged 19 years and older who received the vaccine compared to the same time last year [14]. In New Zealand, influenza vaccinations were 40% higher in 2020 than in 2019. In addition, although it seems unlikely, an influenza vaccine can protect against SARS-CoV-2. Two Italian studies showed that influenza vaccination coverage rates in Italian regions were independently and negatively associated with SARS-CoV-2 seroprevalence, hospitalizations for COVID-19 symptoms, admission to intensive care units, and deaths attributable to COVID-19 [15,16]. (3) Fewer people are tested this year for flu than for COVID-19 and this can lead to hypo-diagnosis of flu infections. People fearing COVID-19 avoided visiting a physician to get a diagnosis. WHO Director-General Dr. Tedros Adhanom Ghebreyesus stated that the WHO has observed a “dramatic decrease” in influenza testing in 2020, including a 62% decrease in the number of specimens shipped to WHO reference laboratories. If such a significant decrease in testing results in the inability to fully capture the scope of transmission, it would result in substantial under-reporting [17]. On the other hand comparing South America, which remains a major hotspot for COVID-19, and New Zealand, which is reporting fewer than 10 cases per day, the major difference in the COVID-19 burden seems not to play a significant role in the level of reported influenza activity. Also, as Table 1 depicts, during the 2020-2021 period, the total number of tests for flu was almost double the number of tests performed in the 2019-2020 period. In this regard, the level of SARS-CoV-2 transmission does not appear to have a strong association with seasonal influenza reporting, which provides further indication that the low reported seasonal influenza incidence accurately represents the current level of influenza activity. (4) The virus interference may be another potential factor that could also play an important role in limiting infections. Accumulating data shows that one respiratory virus can block infection with another virus through stimulation of antiviral defenses in the airway mucosa [18,19]. Wu et al. in a recent study reported that a prior rhinovirus infection greatly reduces the chances of contracting influenza A [20]. The reason is that infection with one virus prompts the body’s immune system to release interferon, which blocks replication of all viruses.

Early reports from China suggested that co-infection with other respiratory pathogens was rare. If this is the case, patients positive for other pathogens might be assumed unlikely to have SARS-CoV-2 and vice versa. Data supporting the viral interference hypothesis also come from the 2009 influenza A pandemic. Accumulating test results from several European countries indicated that the annual autumn rhinovirus epidemic interrupted and delayed the transmission of the emerging influenza virus [21,22]. Since 2009, analyzes of co-detections of common respiratory viruses, including rhinovirus and influenza A virus or rhinovirus and respiratory syncytial virus have shown that co-detections are significantly lower than it would be expected by chance alone, supporting the viral interference hypothesis [23-28].

The low number of flu infections during 2020-2021 means that there is a slow build-up of susceptibility to influenza viruses and when control measures for COVID-19 are lifted, the number of immunized people will be very low. It is a real possibility that we will see increased outbreaks of endemic infections in the coming years. Some of those infections could be more severe than normal, again because of weaning immunity. Using models fit to historic cases of influenza and RSV, some investigators project large future outbreaks of both diseases. These outbreaks may occur following a period of extended NPIs as soon as these measures are raised or relaxed. Preliminary results for influenza suggest outbreaks may occur outside of the typical season coinciding with the end of the control period, but also outbreaks may occur several years after initial NPIs were put into place [29].

As winter in the southern hemisphere precedes the winter of the northern hemisphere, we usually use the experience and the data collected from the influenza season of the southern hemisphere to predict and forecast the upcoming northern hemisphere influenza season, i.e., selection of influenza virus strains to be included in the seasonal influenza vaccine that will be used for immunization in the northern hemisphere. However, the lack of such data from both hemispheres during 2020-2021 makes it difficult to predict and forecast not only the severity of the expected come back of seasonal influenza but also to prepare the appropriate flu vaccine. At the same time, with fewer virus particles circulating in the world, there is less chance of an upcoming mutation, so it is possible that the 2021-2022 vaccine will prove more effective.

For the time being, the lesson we have learned from the current COVID-19 pandemic is that NPIs have proven effective in reducing the spread not only of SARS-CoV-2 but also of many other directly transmitted respiratory infections in many contexts. Policy measures including mass lockdown, social distancing, school closures, travel restrictions, and the use of masks in public spaces being implemented to reduce the transmission of SARS-CoV-2 may also reduce the transmission of flu and other respiratory infections [30].

Limitations

This study has some limitations. The search was limited to reports from three only data sources (WHO, CDC, and ECDC) which cover the majority of worldwide cases, but other data sources could also be used in the study. In addition, as COVID-19 is an ongoing pandemic and some data are still lacking a future data analysis by the end of the pandemic could give us more accurate information regarding the impact of the COVID-19 pandemic on seasonal influenza.

## Conclusions

Despite the millions of infected and dead people worldwide, COVID-19 could serve as a challenge from an epidemiological point of view in order to extract useful information in many contexts. During the COVID-19 pandemic and for two consecutive years (2020 and 2021), all directly transmitted respiratory infections almost disappeared. Collected data during this period showed that NPIs might have played a key role in this phenomenon and could be used successfully in the future for similar pandemic control.
